# A Comparative Study and Introduction of a New Heat Source Model for the Macro-Scale Numerical Simulation of Selective Laser Melting Technology

**DOI:** 10.3390/ma19030480

**Published:** 2026-01-25

**Authors:** Hao Zhang, Shuai Wang, Junjie Wang, Zhiqiang Yan

**Affiliations:** 1 School of Physics and Electronic Information, Yan’an University, Yan’an 716000, China; haozhang043610@163.com; 2 Shaanxi Institute of Technology, Xi’an 710300, China; 13227071835@163.com; 3 Key Laboratory of Green and Intelligent Development and Efficient Utilization of Strategic Mineral Resources of Xinjiang Production and Construction Corps, School of Mechatronics and Automation Engineering, Xinjiang University of Technology, Hotan 848000, China; 4 Key Laboratory of Road Construction Technology and Equipment of MOE, Chang’an University, Xi’an 710064, China

**Keywords:** selective laser melting, finite element, dynamic heat source, temperature field, molten pool size

## Abstract

**Highlights:**

**What are the main findings?**

- A three-dimensional transient heat transfer finite element model was constructed using APDL to investigate the temperature distribution and molten pool characteristics under four heat source models.
- A dynamic model combining Gaussian surface and rotating body heat sources was proposed, enabling dynamic allocation of laser energy absorption ratios between the powder surface layer and substrate depth.
- Predicted values from the dynamic heat source exhibit relative errors of only 1.0% (width) and 5.5% (depth) compared to experimental data, demonstrating high prediction accuracy.

**What are the implications of the main findings?**

- Resolves the issue of traditional surface/volume heat source models overestimating melt pool width and underestimating melt pool depth, enhancing the reliability of SLM numerical simulations.
- Provides an effective simulation method for temperature field and melt pool evolution under different laser parameters, enriching SLM heat source modeling theory.
- High-precision melt pool prediction offers critical theoretical support for SLM process parameter optimization and porosity defect suppression.

**Abstract:**

Selective Laser Melting (SLM), as a common metal additive manufacturing (AM) technology, achieves high-precision complex part formation by layer-by-layer melting of metal powder using a laser. However, the dynamic behavior of the melt pool during the SLM process is influenced by the heat source model, which is crucial for suppressing porosity defects and optimizing process parameters, directly determining the reliability of numerical simulations. To address the issue of traditional surface heat source models overestimating the melt pool width and volume heat source models underestimating the melt pool depth, this study constructs a three-dimensional transient heat conduction finite element model based on ANSYS Parametric Design Language (APDL) to simulate the evolution of the temperature field and melt pool geometry under different laser parameters. First, the temperature fields and melt pool morphology and dimensions of four heat source models—Gaussian surface heat source, volumetric heat source models (rotating Gaussian volumetric heat source, double ellipsoid heat source), and a combined heat source model—were investigated. Subsequently, a dynamic heat source model was proposed, combining a Gaussian surface heat source with a rotating volumetric heat source. By dynamically allocating the laser energy absorption ratio between the powder surface layer and the substrate depth, the influence of this heat source model on melt pool size was explored and compared with other heat source models. The results show that under the dynamic heat source, the melt pool width and depth are 128.6 μm and 63.13 μm, respectively. The melt pool width is significantly larger compared to other heat source models, and the melt pool depth is about 17% greater than that of the combined heat source model. At the same time, the predicted melt pool width and depth under this heat source model have relative errors of 1.0% and 5.5% compared to the experimental measurements, indicating that this heat source model has high accuracy in predicting the melt pool’s lateral dimensions and can effectively reflect the actual melt pool morphology during processing.

## 1. Introduction

Selective Laser Melting (SLM) is a widely used metal additive manufacturing (AM) technology that employs a high-energy laser beam to selectively scan pre-deposited metal powder, enabling the layer-by-layer fabrication of fully dense metal components [1,2]. Compared with other metal AM technologies such as electron beam melting (EBM), directed energy deposition (DED), and binder jetting, SLM offers superior dimensional accuracy and microstructural control, making it particularly suitable for producing components with complex geometries and fine features. However, this high precision is achieved at the expense of higher residual stresses, relatively limited build size, and greater sensitivity to process parameters. Nevertheless, the unique manufacturing mechanism of SLM allows for the production of intricate and customized parts while shortening design-to-product cycles and reducing material waste and tooling requirements [3,4]. As a result, SLM has been widely adopted in high-performance sectors such as aerospace, automotive engineering, and biomedical applications, where stringent requirements on geometric accuracy, mechanical performance, and material utilization must be simultaneously satisfied [5,6,7,8]. However, this process is characterized by steep temperature gradients and complex thermal dynamics, which can lead to defects including part deformation, balling phenomena, poor surface roughness, and porosity [9,10,11]. In response to these challenges, a growing body of research has focused on enhancing forming quality through both experimental investigations and simulation-based approaches. Given the difficulties, time requirements, and high costs associated with in situ monitoring and process optimization via experimental methods, process simulation has emerged as a preferred strategy for analyzing temperature evolution and developing regulatory techniques for process configuration optimization.

For example, Su et al. developed a three-dimensional transient temperature field model employing ANSYS finite element analysis (FEM) software to compare the thermal behaviors of two representative damping alloys fabricated via SLM technology [12]. This model integrated the thermos-physical properties and latent heat associated with phase transformations of the materials. Subsequently, this study examined the temperature distribution, temperature gradients, and cooling rates of the two alloys during both horizontal and vertical deposition processes at varying scanning speeds. To explore the size-dependent effects on residual stress in annular components produced by SLM, Yan et al. constructed a three-dimensional nonlinear FEM model based on sequentially coupled thermal-structural field analysis, utilizing a moving Gaussian surface heat source [13]. Furthermore, the same FEM model was employed by the authors to evaluate the thermal behavior of SLM-fabricated parts with diverse geometric features [14]. In investigating the impact of thermal cycling on residual stress in alloy components manufactured through SLM, Fang et al. implemented a thermal stress analysis model within the ABAQUS finite element multi-field simulation environment [1]. This approach incorporated laser directional movement and real-time powder layer addition through the use of the DFLUX heat source subroutine and model change techniques. Additionally, Liu et al. studied the effects of the track length and track number on the evolution of the molten pool characteristics of SLMed Al alloy, where a Gaussian heat source model was used to establish the FEM framework [15]. Li et al. investigated the temperature field and the stress fields of a multilayer metal powder under the point exposure scanning mode using the numerical method, which considered the latent heat of phase change, temperature-dependent physical parameters, convective heat transfer, among other factors [16]. Bai et al. studied the effects of printing turn angle on structural deformation and stress in the SLM process using the numerical modeling method [17]. Collectively, these studies demonstrate that macroscopic numerical simulation methods are highly effective for analyzing temperature and stress evolution patterns under varying process parameters, scanning strategies, and geometric features. Nonetheless, this body of work also highlights a critical challenge: enhancing model fidelity to ensure the accurate capture of key physical characteristics inherent to the SLM forming process.

During the SLM process, the laser interacts with multiple material phases, including powder, liquid, and solid states, resulting in a highly complex heat absorption behavior characterized by strong spatial and temporal heterogeneity in laser–material interaction [18]. In the powder state, the intricate morphology of the powder bed induces mutual shielding, multiple scattering, and multiple reflections of laser rays among the particles, leading to volumetric and non-uniform energy deposition. Furthermore, variations in material composition, layer thickness, and particle size distribution within the powder bed further intensify this heterogeneity by altering local optical penetration depth and effective absorptivity. Additionally, laser parameters influence the morphology of the molten pool, thereby modifying the local surface condition and absorption behavior. Upon laser irradiation, the powder undergoes a phase transition into the liquid state. In this phase, Marangoni convection, recoil pressure, and surface deformation give rise to a dynamically evolving and highly irregular molten pool geometry, which continuously changes the local incidence angle, reflectivity, and energy coupling of the laser. Once the laser beam is withdrawn, the molten pool rapidly cools and solidifies. When the laser subsequently interacts with the solid phase, energy is predominantly absorbed at the surface and conducted inward, with the effective absorptivity again depending on surface roughness, layer thickness, and laser parameters. Therefore, because laser energy absorption varies with material phase, surface morphology, and time during scanning, the development of a heat source model that can represent these coupled and evolving physical processes is essential for enhancing the fidelity of numerical simulations.

At present, numerical simulations of SLM are mainly classified into macro-scale and meso-scale models. Macro-scale models are capable of predicting the thermal field characteristics (such as melt pool temperature and melt pool dimensions) and stress-field characteristics (such as deformation and thermal stress) during the SLM process. These micro- and meso-scale features have a significant influence on macroscopic functional properties, including surface quality, corrosion resistance, and mechanical strength. Recent multiscale surface studies have demonstrated that nano- and micro-scale topographical complexity is strongly correlated with functional surface responses, indicating that textures formed at small length scales can significantly affect the overall performance of engineering surfaces [19]. From this perspective, macro-scale thermal simulations should be regarded not only as tools for predicting temperature fields and melt pool dimensions, but also as a means of describing the energy distribution and the macroscopic functional performance of additively manufactured parts. The main heat source models for macro-scale numerical simulation adopted by researchers are the surface heat source, the volumetric heat source, and the equivalent body heat source model. For example, Li et al. proposed a hybrid heat source model which took the surface and volumetric heat flux absorption into consideration, where the surface heat source was used to irradiate the solid and liquid material and the volumetric heat source was utilized to irradiate the power material [20].

Vanini et al. employed a conical Gaussian volumetric heat source exhibiting linear attenuation along the *z*-axis to examine variations in melt pool characteristics during the Selective Laser Melting (SLM) process of Ti-6Al-4V alloy [21]. Ivanov and collaborators utilized a simplified, uniformly distributed heat source in conjunction with selective mesh refinement techniques to simulate macroscopic temperature fields in additive manufacturing, thereby achieving a reduction in computational time [22]. Chen developed a three-dimensional finite element model for the SLM processing of copper alloys, substituting the laser melting of powder material with a Gaussian surface heat source and integrating neural networks to predict melt pool temperature and dimensions [23]. While Gaussian surface and uniform heat sources offer computational simplicity and enhanced processing speed, they compromise the accuracy of melt pool size predictions. Luo et al. conducted a comparative analysis between Gaussian surface and double ellipsoidal heat sources to assess their suitability in SLM process simulations, concluding that the double ellipsoidal model yielded results with higher fidelity and better agreement with experimental data [24]. He and colleagues introduced an inverse modeling approach grounded in Bayesian algorithms to optimize heat source parameters by calibrating them against observed melt pool morphologies [25]. Tran investigated the influence of powder size distribution on laser energy propagation along the powder bed depth, proposing a volumetric heat source model incorporating dynamic absorptivity [26]. Furthermore, several studies have explored adjustments to heat source models based on powder absorptivity and melt pool geometry [27,28]. The extensive body of literature on heat source modeling reveals that these models are generally simplistic and depend heavily on empirical parameters such as laser absorptivity. In practical applications, a single heat source model seldom captures the complex laser–material interactions accurately, as laser absorptivity varies spatially within the powder bed and with depth, complicating real-time control. Additionally, precise localization of the laser-induced thermal load remains challenging. Consequently, simulations employing these heat source models inherently exhibit discrepancies relative to actual processing conditions. It is evident that the aforementioned heat source models feature relative simplicity and rely on empirical parameters (e.g., laser absorptivity). Real-time regulation of the spatiotemporal variations in laser absorptivity with respect to the powder bed’s position and depth poses a common challenge, and the precise location of laser thermal loading also cannot be identified accurately. Consequently, the simulation outcomes derived from such models exhibit intrinsic deviations relative to actual processing conditions.

To address this problem, two related works were conducted in this paper. First, four typical heat source models (planar Gaussian heat source model, the rotating Gaussian volumetric heat source model, the double ellipsoidal heat source model, and the combined heat source model) programmed as user-defined subroutines are implemented into a 3D nonlinear transient heat transfer finite element model developed in ANSYS, where temperature distribution, molten pool dimensions, and molten pool shape are analyzed and compared. Then a new dynamic heat source model is designed to clearly simulate the laser interacting with multiple material phases. Next, the temperature evolution and molten pool characteristic under this heat source model is analyzed and compared with other heat source models to demonstrate its superiority in macro-scale numerical simulation. Finally, experiments are conducted to verify the effectiveness of the proposed new heat source model.

The rest of this paper is organized as follows: Section 2 introduces the mathematical characterization methods of four heat source models (Gaussian surface heat source, rotating Gaussian volumetric heat source, double ellipsoid heat source, and combined heat source model). Section 3 mainly provides detail on the thermal analysis and simulation design. In Section 4, the temperature evolution and molten pool characteristic under these four heat source models are analyzed and compared, and a new dynamic heat source was proposed. Section 5 provides the experimental details and comparison between the measured molten pool size and simulated size. Section 6 summarizes the conclusions and limitations, and highlights possible future works.

## 2. Mathematical Representation of Heat Source Models

Compared to measuring or monitoring the temperature evolution during the SLM process, finite element simulation has been proven to be a reliable method for comprehensively understanding the temperature evolution, melt pool geometry, and residual stress in SLM forming [29]. Establishing a high-fidelity numerical model is key to accurately predicting temperature changes and residual stress distribution during the SLM process. As the foundation of numerical simulation studies, the heat source model’s rationality and parameter accuracy directly affect the reliability of predictions regarding melt pool geometry, temperature gradient fields, and thermal cycling history [30,31,32]. Below, the mathematical representations of four types of heat sources are presented: planar Gaussian heat source, volumetric distribution heat source models (rotating Gaussian volumetric heat source model, double ellipsoidal distribution heat source model), and the combined heat source model. This lays the groundwork for subsequent multi-set SLM numerical simulations based on different heat sources and data extraction.

### 2.1. Planar Gaussian Heat Source Model

The planar distributed heat source model can better fit the actual power distribution of the heat source, making it closer to the real laser heat source [33]. Among them, the Gaussian surface heat source describes the energy distribution of the heat source, where the energy input follows a Gaussian function distribution within a certain circular area. The heat flux distribution at the heating point is described by a Gaussian function as follows:

(1) $$q(r)=q_{m}\exp(-\frac{3r^{2}}{r_{H}^{2}})$$

q(r) denotes the heat flux density at a distance from the center of the heat source, $q_{m}$ denotes the maximum heat flux density at the center of the heat source, and $r_{H}$ denotes the radius of the heat source. Assuming the input power of the laser is *Q*:

(2) $$\begin{array}{l} Q=\int_{0}^{\infty }q(r)2\pi r\;dr\\ =q_{m}\int_{0}^{\infty }\exp(-\frac{3r^{2}}{r_{H}^{2}})2\pi r\;dr\\ =\frac{q_{m}\pi r_{H}^{2}}{-3}\int_{0}^{\infty }\exp(-\frac{3r^{2}}{r_{H}^{2}})\;d(-\frac{3r^{2}}{r_{H}^{2}})\\ =\frac{q_{m}\pi r_{H}^{2}}{3} \end{array}$$

(3) $$q_{m}=\frac{3Q}{\pi r_{H}^{2}}$$

They can be organized as follows:

(4) $$q(r)=\frac{3Q}{\pi r_{H}^{2}}\exp(\frac{3r^{2}}{r_{H}^{2}})$$

The heat flux density under a planar Gaussian heat source exhibits a symmetrical distribution and has strong applicability within a certain range of laser scanning speeds, allowing it to match actual heat source models. However, for cases where a deeper melt pool is required, as well as for excessively high or low scanning speeds, the fitting error of the planar Gaussian heat source model is relatively large.

### 2.2. Volumetric Heat Source Model

The volumetric distributed heat source is suitable for scenarios with high energy density SLM forming and high requirements for the accuracy of molten pool fitting. A three-dimensional thermal source model is used to meet the needs of numerical simulation. The commonly used volumetric heat source models include the rotating Gaussian volumetric heat source model and the double ellipsoidal distributed heat source model [34]. Compared to the molten pool formed in the actual process, it is found that the inverted cone shape better matches the characteristics of the molten pool, which corresponds to the rotating Gaussian volumetric heat source model. Its heat flux density distribution satisfies

(5) $$q(r,h)=Q\exp\left[\frac{-3c_{s}}{\log(\frac{H}{z})}r^{2}\right]$$

(6) $$c_{s}=\frac{3}{R^{2}}$$

The total heat flow input from the heat source is equal to the laser heat input power, which means that

(7) $$\begin{array}{l} Q=\int_{0}^{H}\int_{0}^{2\pi }\int_{0}^{R}q(r,h)\;drd\theta dh\\ =\int_{0}^{H}2\pi \times \frac{q(0,0)}{2}\times \frac{\log(\frac{H}{h})}{-3c_{s}}\times (e^{-3}-1)dh\\ =\frac{\pi }{3c_{s}}\times q(0,0)\times (1-\frac{1}{e^{3}})\times \int_{0}^{H}\log(\frac{H}{h})dh\\ =\frac{\pi H}{3c_{s}}\times (1-\frac{1}{e^{3}})\times q(0,0) \end{array}$$

They can be organized as follows:

(8) $$q(x,y,z)=\frac{3c_{s}Q}{\pi H(1-\frac{1}{e^{3}})}\exp\left[\frac{-3c_{s}}{\log(\frac{H}{z})}(x^{2}+y^{2})\right]$$

In the formula: q(x,y,z) represents the heat flux density (x,y,z) under local coordinates, *Q* represents the total input power of the laser, *H* represents the height of the heat source, $c_{s}$ represents the opening radius of the heat source, *R* represents the radius of the heat source.

Due to the movement of the laser heat source, the distribution of heat flux density is asymmetric. The heating area at the front end of the volumetric heat source is smaller than that at the rear end. The double ellipsoidal heat source model consists of two parts: the front and the rear. The heat flux distribution of the two parts can be described as follows:

(9) $$q_{f}(x,y,z)=\frac{6\sqrt{3}(f_{f}Q)}{a_{f}b_{h}c_{h}\pi \sqrt{\pi }}\exp(-\frac{3x^{2}}{a_{f}^{2}}-\frac{3y^{2}}{b_{h}^{2}}-\frac{3z^{2}}{c_{h}^{2}}),x\geq 0$$

(10) $$q_{r}(x,y,z)=\frac{6\sqrt{3}(f_{r}Q)}{a_{r}b_{h}c_{h}\pi \sqrt{\pi }}\exp(-\frac{3x^{2}}{a_{r}^{2}}-\frac{3y^{2}}{b_{h}^{2}}-\frac{3z^{2}}{c_{h}^{2}}),x<0$$

The heat input power of the front half of the double ellipsoidal heat source is

(11) $$\begin{array}{l} 2\int_{0}^{\infty }\int_{0}^{\infty }\int_{0}^{\infty }q_{f}(x,y,z)dxdydz\\ =\frac{6\sqrt{3}}{a_{f}b_{h}c_{h}\pi \sqrt{\pi }}\int_{0}^{\infty }\exp{\left(-\frac{3x^{2}}{a_{f}^{2}}\right)}^{2}dx\int_{0}^{\infty }\exp{\left(-\frac{3y^{2}}{b_{h}^{2}}\right)}^{2}dy\int_{0}^{\infty }\exp{\left(-\frac{3z^{2}}{c_{h}^{2}}\right)}^{2}dz\\ =2\times \frac{6\sqrt{3}(f_{f}Q)}{a_{f}b_{h}c_{h}\pi \sqrt{\pi }}\times \frac{a_{f}}{\sqrt{3}}\times \frac{\sqrt{\pi }}{2}\times \frac{b_{h}}{\sqrt{3}}\times \frac{\sqrt{\pi }}{2}\times \frac{c_{h}}{\sqrt{3}}\times \frac{\sqrt{\pi }}{2}\\ =\frac{1}{2}(f_{f}Q) \end{array}$$

Similarly, the thermal input power of the rear half of the double ellipsoid heat source is

(12) $$2\int_{0}^{\infty }\int_{0}^{\infty }\int_{0}^{\infty }q_{r}(x,y,z)dxdydz=\frac{1}{2}(f_{r}Q)$$

At the same time:

(13) $$Q=\frac{1}{2}(f_{f}Q)+\frac{1}{2}(f_{r}Q)=\frac{1}{2}Q(f_{f}+f_{r})$$

(14) $$f_{f}+f_{r}=2$$

In the formula: $f_{f}$, $f_{r}$ represent the proportion of thermal input power for the front and rear sections, respectively, $a_{f}$, $a_{r}$, $b_{h}$, $c_{h}$ represent the semi-axis lengths of the double ellipsoid, respectively. q(x,y,z) represents the heat flux density (x,y,z) under local coordinates, *Q* represents the total input power of the laser. The double ellipsoidal heat source takes into account the effects of heat conduction, heat radiation, and heat convection during the actual forming process, allowing it to better fit the actual laser scanning and reflect the changes in the temperature gradient of the temperature field. The aforementioned heat source model can ensure a certain level of accuracy when simulating SLM forming. The planar distributed heat source has a relatively uniform and stable heat flow distribution, while the volumetric distributed heat source considers the heat input in the depth direction.

### 2.3. Combined Heat Source Model

When using a single heat source, the shape of the simulated molten pool cross-section is relatively fixed, and it overlooks the differences between the surface of the molten pool and the interior, making it difficult to describe the complex thermal behavior during the SLM process. Therefore, it is necessary to consider mixing multiple different heat sources to better match the shape of the molten pool and the internal heat flow distribution. The combined heat source has characteristics of both planar distributed heat sources and volumetric distributed heat sources, which can not only reflect the distribution pattern of surface energy input but also fit well with the heat flow distribution in the depth direction. The combination methods of the combined heat source are quite diverse, usually adjusted and constructed through the geometric shape of the molten pool and the heat flow density distribution of the heat source. This study combines a Gaussian surface heat source with a rotating Gaussian volumetric heat source. The rotating Gaussian volumetric heat source can simulate the influence of heat flow distribution within the powder layer, while the Gaussian surface heat source fits the heat flow variations on the melt pool surface during the SLM process. The total laser energy input power is divided into the surface heat source part and the volumetric heat source part.

(15) $$q_{1}(x,y)=\frac{3Q\alpha }{\pi r_{H}^{2}}\exp(-\frac{3(x^{2}+y^{2})}{r_{H}^{2}})$$

(16) $$q_{2}(x,y,z)=\frac{3c_{s}Q}{\pi H(1-\frac{1}{e^{3}})}\exp\left[\frac{-3c_{s}}{\log(\frac{H}{z})}(x^{2}+y^{2})\right]$$

In the formula: $q_{1}(x,y),\;q_{2}(x,y,z)$ represent the heat flux density distribution of the surface heat source part and the volume heat source part in the local coordinate system. *Q* represents the total input power, *α* and *β* represent the power proportion coefficients of the surface heat source and volume heat source, α+β=1, $r_{H}$ indicates the effective radius of the heat source, *H* represents the height of the heat source, $c_{s}$ represents the opening radius of the heat source.

Establish SLM simulation models for the Gaussian plane heat source, Gaussian rotating body heat source, double ellipsoid heat source, and the combined heat sources of the Gaussian plane and Gaussian rotating body. By comparing aspects such as the temperature field variation characteristics and the dimensions of the melt pool [35], continuously adjust the energy distribution coefficients and shape parameters of the heat sources to improve the fidelity of the simulation model and better guide practical processing [36].

## 3. Establishment of the Finite Element Modeling

**Figure 1:**
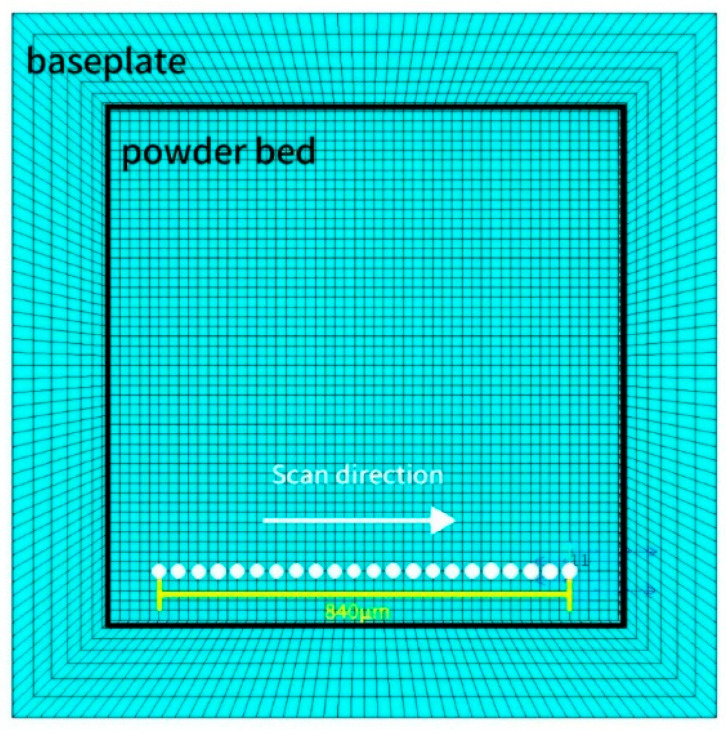
Schematic diagram of single-path scanning.

**Figure 2:**
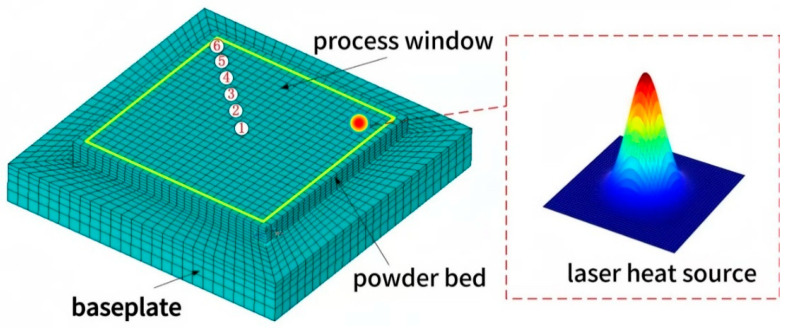
Schematic diagram of grid division model and reference points.

For the numerical simulation of the entire SLM forming process, a flat thin-walled part model was established using the APDL parametric language. The geometric model consists of two parts: the powder bed and the substrate. The substrate measures 1.44 mm × 1.44 mm × 0.2 mm, while the powder bed measures 1.04 mm × 1.04 mm × 0.09 mm. The thickness of a single powder layer is set to 0.03 mm, requiring a total of three layers to be printed. When meshing, it is essential to consider both the solution efficiency and simulation accuracy in the numerical simulation. The mapping method was used for mesh division, with the powder bed mesh size divided into hexahedral eight-node elements of 0.02 mm × 0.02 mm × 0.015 mm. The mesh size in the melt pool region (0.02 mm × 0.02 mm × 0.015 mm) was selected based on our previously published studies on LPBF numerical simulations, which demonstrated that element sizes on the order of 20 μm provide a good balance between accuracy and computational efficiency [13,14]. The substrate mesh is divided into two parts: the area in contact with the powder layer has the same mesh size, while the area away from the powder layer is sparsely divided, with mesh size proportional to the distance from the powder layer. The scanning strategy for the laser heat source employs a raster back-and-forth scanning method, with a single scan path illustrated in Figure 1 Additionally, to better study the thermal evolution of the formed part, six reference points were set at equal distances along the path from the center of the model to the corner edges of the model. The mesh division model and reference point schematic are shown in Figure 2.

**Figure 3:**
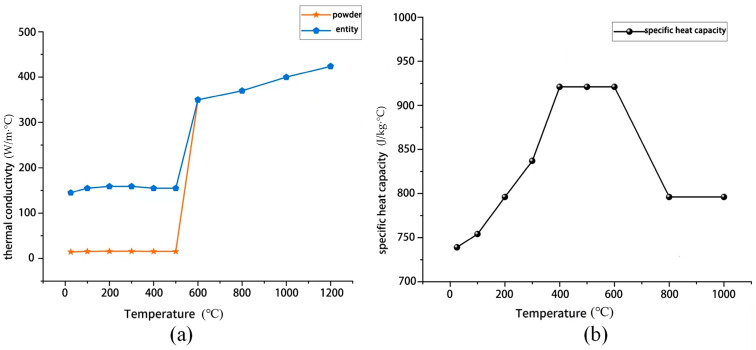
Thermal physical parameters of AlSi_10_Mg material: (**a**) thermal conductivity as a function of temperature; (**b**) specific heat capacity as a function of temperature.

The optimal process parameters for SLM forming have been studied in previous research. In this study, laser power P = 280 W, scanning speed v = 1800 mm/s, and scanning spacing of 0.12 mm were selected as the core process parameters. The theoretical basis for these parameters comes from multi-scale thermodynamic analysis results. When the laser power is below 250 W, insufficient energy input causes the molten pool volume to shrink sharply, resulting in discontinuous spheroidized melt tracks. When the scanning speed exceeds 2000 mm/s, the cooling rate at the solid–liquid interface increases significantly, triggering instability in Marangoni convection. Analysis shows that a continuous and stable molten pool morphology can be achieved at P = 280 W. Additionally, a scanning spacing of 0.12 mm provides an ideal overlap rate between adjacent melt tracks, resulting in a continuous and defect-free molten pool surface morphology. Therefore, this study adopts this set of process parameters. Thermal physical properties have a significant impact on simulation accuracy. The thermal physical parameters of the AlSi_10_Mg material used in this study were all obtained through calculations by JmatPro7.0 software. The density of AlSi_10_Mg is essentially unaffected by temperature and is set at 2680 kg/m^3^. Its thermal conductivity and specific heat capacity vary with temperature as shown in Figure 3. The yellow line represents the thermal conductivity of AlSi_10_Mg in powder form, while the blue line indicates that of AlSi_10_Mg in solid form. It can be observed that prior to the melting point (600 °C), the thermal conductivity of the solid consistently exceeds that of the powder. Due to discontinuous thermal conduction pathways, limited interparticle contact area, and gas-filled voids in the powder state, the effective thermal conductivity of AlSi_10_Mg powder beds is significantly lower than that of bulk material.

**Figure 4:**
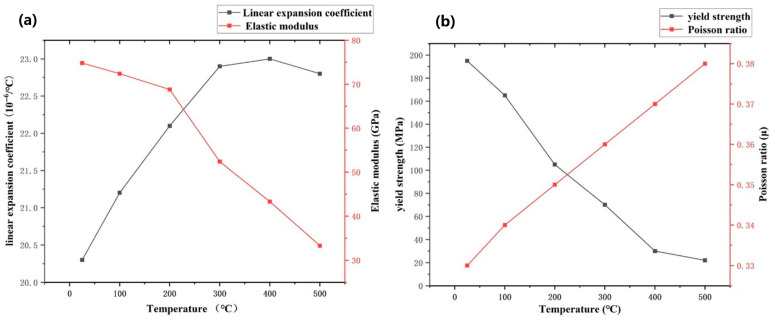
Mechanical property parameters of AlSi_10_Mg material: (**a**) curve of linear expansion coefficient and elastic modulus varying with temperature, (**b**) curve of yield strength and Poisson’s ratio varying with temperature.

There are also some mechanical performance parameters in stress-field analysis, and their values change with temperature. The required mechanical parameters include thermal expansion coefficient, yield strength, elastic modulus, and Poisson’s ratio. The specific variation in these mechanical parameters with temperature is shown in Figure 4.

Based on our team’s previous research, the following assumptions are made for the numerical model in this paper [14]:

(1) During the processing, since the laser penetration depth is approximately equal to the thickness of the powder layer, the heat convection coefficient and heat radiation coefficient between the powder bed surface and the surrounding environment are set as fixed constants;
(2) The powder is considered as an isotropic, continuously uniform medium, with powder particles being uniform-sized and undeformed spheres. During the SLM forming process, the volume of the powder particles remains constant, and heat transfer between the powder voids is not considered;
(3) The effect of the flow of liquid metal in the melt pool on the temperature field during processing is not considered.

In the SLM forming process, the element birth and death technique was employed to simulate the layer-wise filling and melting of metal powder [37,38]. At the beginning of the transient thermal analysis, boundary conditions corresponding to the preheated substrate were applied, and a steady-state thermal solution was first performed to obtain an initial temperature field for the transient simulation. Subsequently, transient thermal analysis was carried out to capture the layer-by-layer thermal evolution. Each laser load step was subdivided into two sub-steps to ensure sufficient temporal resolution of the moving heat source. The laser step length was set equal to the in-plane mesh size (PD = 0.02 mm), and the exposure time per step was defined as $T_{1}=PD/v$. For the present processing condition (v = 1800 mm/s), this gives $T_{1}=1.11\times 10^{-5}$ s, resulting in a time increment of $\Delta t=T_{1}/2=5.56\;$ per sub-step. This time-stepping strategy allows the steep thermal gradients induced by the moving laser to be accurately resolved while maintaining numerical stability. In the transient thermal analysis, an initial temperature of 25 °C was applied as the boundary condition to represent the system state after substrate preheating, and a steady-state analysis was first conducted to obtain the initial temperature field for the numerical model. During the transient thermal simulation, both the substrate temperature and the ambient temperature were set to 65 °C, and each load step was further subdivided into two sub-steps. An inter-layer cooling time was defined within each layer cycle to simulate the time required for powder spreading and control-system adjustments during the single-layer masking process. After completion of the sample fabrication, the boundary conditions were redefined and the ambient temperature was changed to room temperature to simulate the cooling of the formed part. The detailed transient solver and time-stepping parameters are summarized in Table 1.

## 4. Results and Discussion

### 4.1. Temperature Field

**Figure 5:**
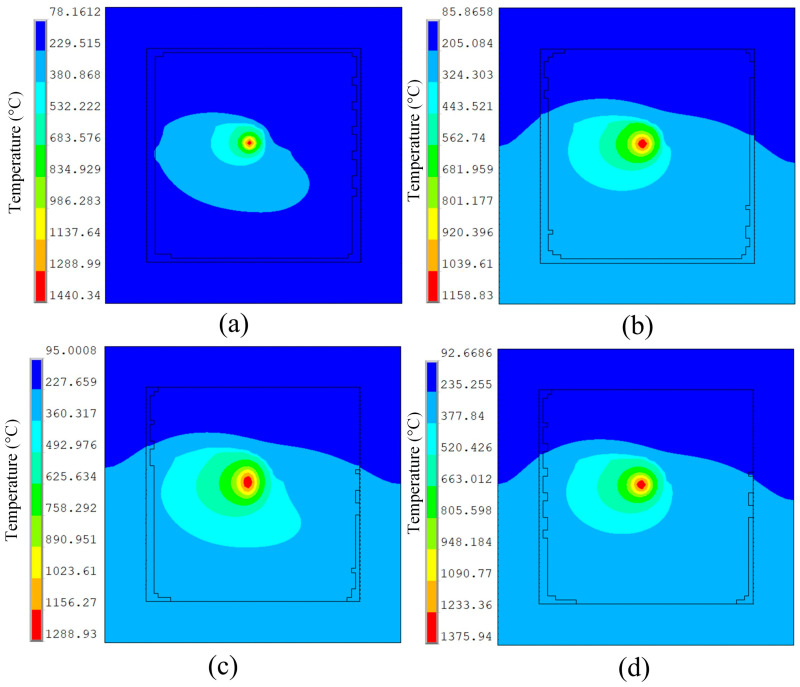
Temperature cloud maps under different heat source models: (**a**) Gaussian surface heat source, (**b**) Gaussian volumetric heat source, (**c**) double ellipsoidal heat source, (**d**) combined heat source.

To ensure physical consistency, the boundary conditions for surface heat transfer, interlayer cooling time, and ambient temperature were set according to the experimental conditions. Meanwhile, a macro-scale LPBF modeling framework was adopted to balance computational efficiency and physical fidelity, which is known to accurately capture global temperature evolution and heat accumulation during multi-layer SLM. Using post-processing commands, temperature field characteristic data from four different heat sources were extracted for comparison. A comparative analysis was conducted on the temperature field at the middle position of the fifth melt channel in the molded part. Figure 5 shows the temperature field cloud maps at this position for different heat sources. Figure 5a–d correspond to the temperature cloud maps under Gaussian surface heat source, Gaussian volumetric heat source, double ellipsoidal heat source, and combined heat source, respectively.

Figure 5a shows the temperature contour map of the Gaussian surface heat source. It can be observed that under this heat source model, the heat-affected zone (HAZ) is elliptical in shape. The area of the HAZ at the front end of the heat source is much smaller than that at the rear end. This is influenced by the scanning strategy; the locations where the laser has scanned have not fully cooled down, and the previous melt tracks also affect the size of the HAZ. The temperature isotherms at the front end of the heat source are relatively dense, indicating a steep temperature gradient. Figure 5b,c both show temperature contour maps for volumetric heat sources. In Figure 5b, the temperature gradient at the center of the heat source forms a quadrilateral shape, while in Figure 5c, the temperature gradient at the center matches a double ellipsoid distribution. Compared to the temperature contour map of the planar heat source, it is evident that the volumetric heat source results in a larger HAZ. This is because, under the volumetric heat source, the energy distribution is mainly along the depth direction of the powder bed, causing more powder nodes to be subjected to thermal loading. The cumulative thermal effect between nodes is significant. Additionally, the volumetric heat source model accounts for laser energy penetration, transferring heat to the substrate. Since the substrate’s thermal conductivity is much higher than that of the powder material, the HAZ also extends into the substrate, increasing its overall area. In contrast, the Gaussian surface heat source acts on the surface of the powder bed, where air convection, conduction, and radiation are more intense, causing a large amount of heat to dissipate into the surrounding environment. Meanwhile, the powder material has poor thermal conductivity, resulting in a smaller HAZ. Figure 5d shows the temperature contour of the combined heat source model, which is formed by superimposing the Gaussian surface heat source and the volumetric heat source. The distribution characteristics of the HAZ combine the features of both the surface heat source model and the volumetric heat source model. The area of the HAZ is larger than that of the planar distributed heat source, while the peak temperature at the center of the heat source is significantly higher compared to the volumetric heat source model, and the temperature gradient is also reduced.

**Figure 6:**
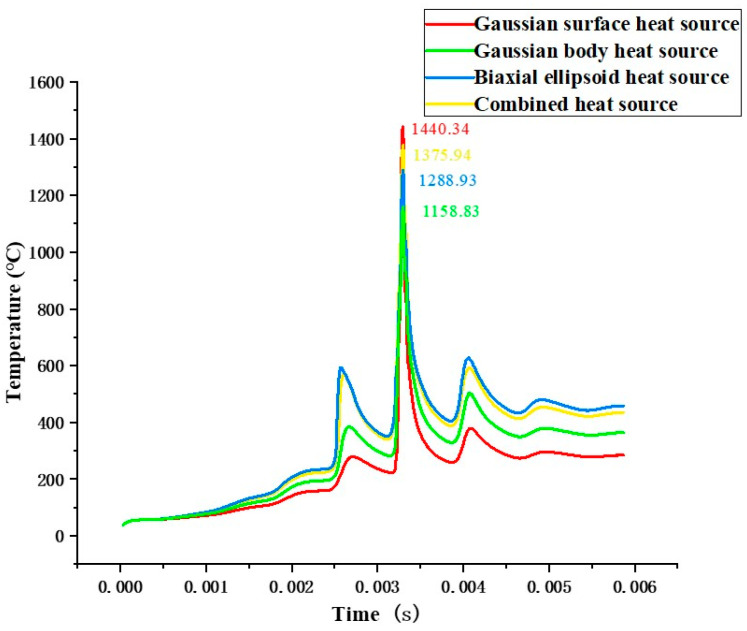
Temperature variation curve of node 12920 over time under different heat sources.

Figure 6 shows the temperature variation over time at the center of the fifth weld pool, specifically at node 12920, under different heat sources. Comparing the four curves in the figure, it can be observed that all curves follow a similar trend: a rapid rise to the peak temperature followed by a gradual decline. This indicates that different heat sources undergo a similar thermal loading process at the same location. The planar Gaussian heat source model exhibits the highest peak temperature of 1440.34 °C, which is significantly higher than the other heat sources, and it also produces the largest temperature gradient. The peak temperatures under the two volumetric heat sources are lower than those of the other two heat sources, with the Gaussian volumetric heat source and the double ellipsoidal heat source reaching peaks of 1158.83 °C and 1288.93 °C, respectively. The combined heat source, which integrates features of both the surface and volumetric heat source models, results in an HAZ larger than that of the planar distributed heat source. Additionally, the peak temperature at the heat source center is significantly increased compared to the volumetric heat source models, reaching 1375.94 °C, while also reducing the temperature gradient.

Observing the curves in Figure 6 reveals that at the onset of laser scanning, the temperature rise trends of the four heat sources at the molten pool node are nearly identical. However, the molten pool formed under the dual ellipsoidal heat source and the combined heat source exhibits the highest peak temperature, indicating that the energy density concentrated at the molten pool center is greater for these two heat sources. Additionally, the rate at which the temperature curves decrease after the laser scanning is slower than that of the other two heat sources. This suggests that the double ellipsoid and combined heat sources have a stronger heat accumulation effect, enabling them to fully melt the metal powder material and reduce the temperature gradient, thereby weakening thermal stress. The melting point of the aluminum alloy material is 600 °C. The Gaussian surface heat source curve maintains a temperature above the melting point for the shortest duration, meaning the Gaussian surface heat source has the shortest molten pool lifetime. In contrast, the volumetric heat source and combined heat source curves, due to their energy distribution in the depth direction and mutual influence between adjacent nodes, accumulate heat, resulting in a longer molten pool existence time. Although convective and radiative heat losses vary in practice, heat conduction into the solidified region and substrate dominates the thermal response when the laser penetration depth is comparable to the powder layer thickness. Therefore, using constant surface heat transfer coefficients mainly affects near-surface peak temperature and cooling rate, but has limited influence on the global thermal accumulation and inter-layer thermal cycling observed in Figure 5 and Figure 6.

**Figure 7:**
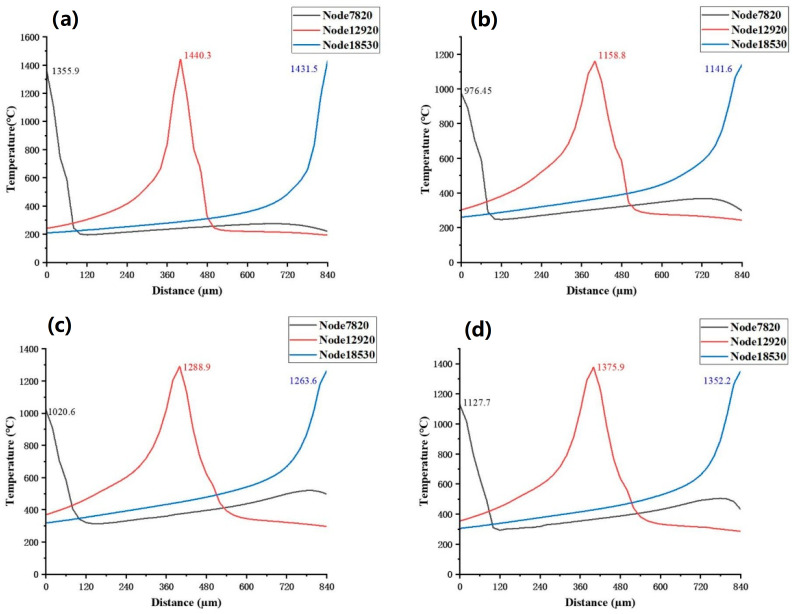
Temperature variation curves along the scanning paths at nodes 7820, 12920, and 18530 with respect to distance: (**a**) Gaussian surface heat source, (**b**) Gaussian volumetric heat source, (**c**) double ellipsoidal heat source, (**d**) combined heat source.

Temperature variation curves along the scanning path were extracted at three points of the fifth weld bead: the start point at node 7820, the midpoint at node 12920, and the end point at node 18530. Figure 7a–d correspond to the Gaussian surface heat source, Gaussian volumetric heat source, double ellipsoidal heat source, and combined heat source, respectively. It can be observed that the Gaussian surface heat source produces the highest peak temperatures at all nodes, especially at the start and end points of the scanning path, with values of 1355.9 °C and 1431.5 °C, respectively. This indicates that the Gaussian surface heat source has the most pronounced localized heating effect. Compared to the other heat sources, the Gaussian volumetric heat source shows a more uniform temperature variation, with the lowest peak temperatures at all nodes; the peak temperature at the midpoint is 1158.8 °C. The overall trend of the double ellipsoidal heat source curve is similar to that of the rotating Gaussian volumetric heat source. For the combined heat source, the temperature distribution is more gradual, with smaller temperature differences between nodes, demonstrating its strong capability for uniform overall processing.

Among the four different heat source models, the temperature at node 7820 is the lowest, and the temperature curves at nodes 12920 and 18530 change more gradually. Since the heat accumulation of each heat source is more significant in the middle region of the scanning path, node 12920 represents the peak temperature position along the entire melt pool. The temperature curve of the double ellipsoid heat source shows a slope change more similar to that of the combined heat source, but overall, the combined heat source performs better than the double ellipsoid heat source, with peak temperatures at all three nodes exceeding those of the double ellipsoid heat source.

### 4.2. The Shape and Size of the Molten Pool

In the SLM process, the shape and size of the melt pool will directly affect the melting and cooling of the powder material, thereby influencing the microstructure and mechanical properties of the formed parts [39]. The width of the melt pool is the lateral span of the melt pool on the surface of the powder bed; this parameter indicates the range of the lateral distribution of laser energy and also determines the overlap rate between adjacent melt tracks. The overlap of adjacent melt tracks can effectively enhance the density of the parts. The depth of the melt pool is the vertical distance from the surface of the powder bed to the bottom of the melt pool; this value reflects the penetration capability of the laser beam. The melt pool depth must ensure sufficient metallurgical bonding between the processing layer and the previous layer to ensure the strength of the parts.

**Figure 8:**
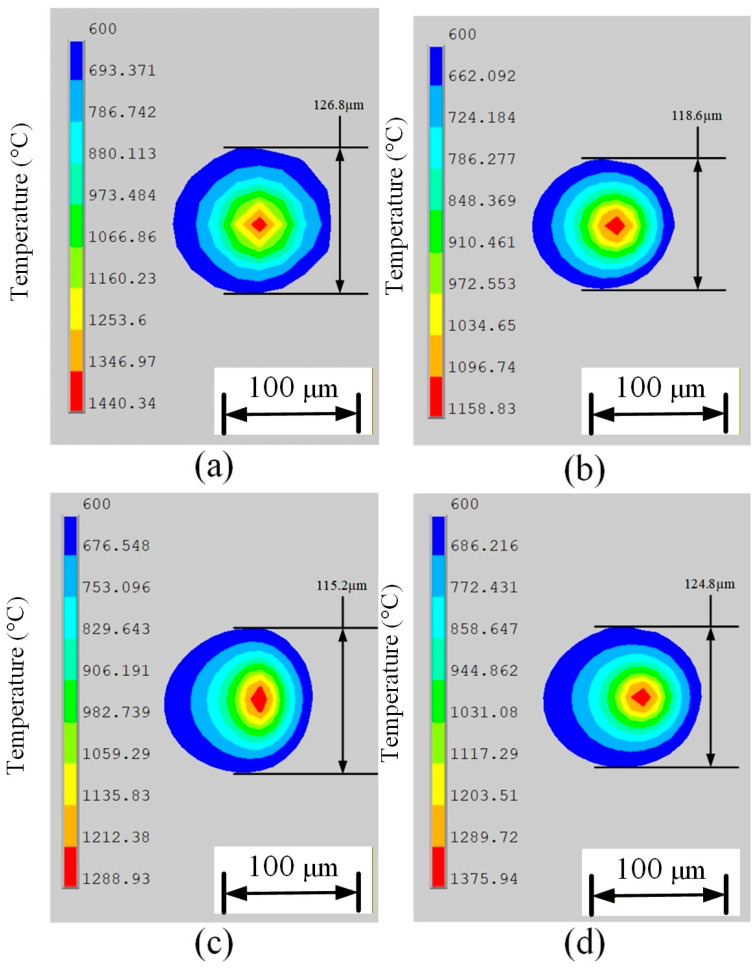
Melt pool morphology and melt pool width under different heat source models: (**a**) Gaussian surface heat source, (**b**) Gaussian volumetric heat source, (**c**) double ellipsoidal heat source, (**d**) combined heat source.

**Figure 9:**
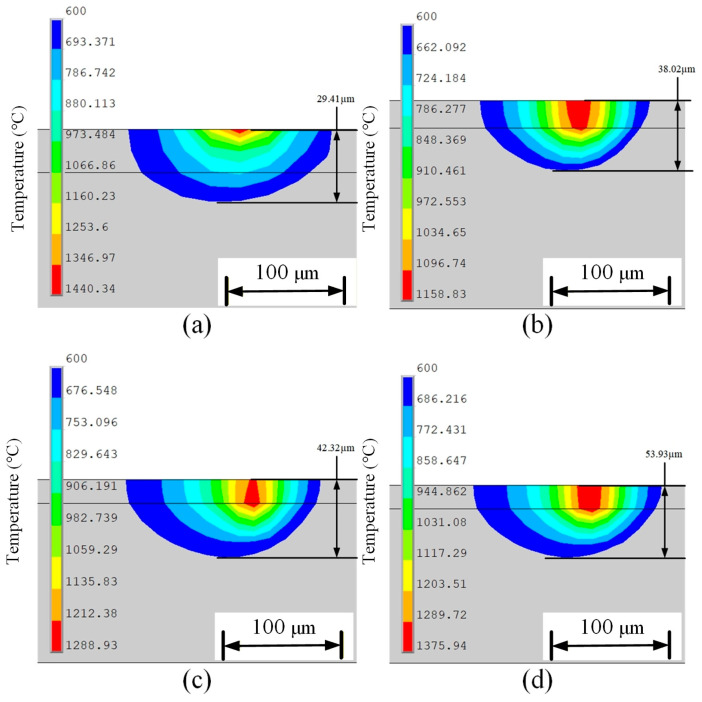
Longitudinal cross-section of the melt pool under different heat source models: (**a**) Gaussian surface heat source, (**b**) Gaussian volumetric heat source, (**c**) double ellipsoidal heat source, (**d**) combined heat source.

The powder bed was modeled as an equivalent isotropic continuum with effective thermophysical properties, which enables the element birth–death technique to simulate powder spreading and melting. While this approach smooths particle-scale thermal gradients, it reliably captures the macroscopic melt pool geometry and penetration depth; thus, the differences in melt pool width and depth in Figure 8 and Figure 9 mainly reflect the energy-deposition characteristics of different heat source models. Figure 8 shows the shape of the melt pool and the width of the melt pool at the midpoint of the fifth melt track. In Figure 8a, the melt pool shape with a Gaussian surface heat source is closer to a circular shape, with the heat source center appearing as a perfect square, distributing evenly outward, and the heat concentrated in the middle part of the melt pool. In Figure 8b, the melt pool shape with a Gaussian body heat source is slightly flattened, with the heat source center appearing as a diamond shape. The isotherms at the front end of the heat source are evenly distributed, while the heat at the rear end diffuses more laterally. Figure 8c shows the melt pool with a double ellipsoidal heat source, which has an elliptical outline, and the heat distribution expands uniformly laterally. In Figure 8d, the melt pool with a combined heat source is wide and flat elliptical, indicating that the energy distribution of the heat source is uniform in both lateral and vertical directions.

Regarding the width of the melt pool, the size of the Gaussian surface heat source is 126.8 μm. Due to the concentration of heat at the surface of the Gaussian surface heat source, the melt pool width is larger. The melt pool width with the Gaussian body heat source is 118.6 μm, which is narrower compared to the Gaussian surface heat source, indicating that the heat diffusion of the Gaussian body heat source into deeper layers reduces lateral expansion. The melt pool width with the double ellipsoidal heat source is 115.2 μm, which is smaller than the other three heat source models. The combined heat source integrates the characteristics of both the Gaussian surface heat source and the Gaussian body heat source, resulting in a melt pool width of 124.8 μm. The penetrating effect of the Gaussian body heat source causes the nodes in the depth direction to influence each other, which also enhances the concentration of heat at the surface of the melt pool, leading to a uniform heat distribution and an increase in melt pool width.

Figure 9 shows the cross-sectional view along the *y*-axis at the midpoint of the melt pool. In Figure 9a, the melt pool depth for the Gaussian surface heat source is 29.41 μm, which is the smallest melt pool depth. The area of the red high-temperature region is also the smallest. The melt pool expands more laterally than longitudinally, and the HAZ in the depth direction is small. This indicates that the heat transfer from the Gaussian surface heat source to the powder layer is limited, which may lead to insufficient metallurgical bonding between formed layers, thereby reducing the strength of the formed part. Figure 9b shows the melt pool depth for the Gaussian volumetric heat source is 38.02 μm, with a larger melt depth size. The heat distribution penetrates deeply into the material, and the red high-temperature region extends significantly in the longitudinal direction, demonstrating that the heat can effectively diffuse deeper, and the laser has a certain penetration capability. Figure 9c shows the melt pool cross-section for the double ellipsoid heat source, with a melt pool depth of 42.32 μm. Although the melt pool depth of the volumetric distribution heat source is much greater than that of the Gaussian surface heat source, and its red high-temperature region is symmetrically distributed, the area is smaller compared to the Gaussian volumetric heat source. This indicates that the penetration ability of the double ellipsoid heat source in the depth direction of the powder layer is inferior to that of the Gaussian volumetric heat source. Figure 9d shows the melt pool depth for the combined heat source, which is 53.93 μm—the largest melt pool depth. The area of the red high-temperature region is also the largest, with strong heat expansion capabilities both laterally and longitudinally, effectively ensuring interlayer bonding strength. This demonstrates that the combination of the Gaussian surface heat source and the Gaussian volumetric heat source can enhance heat transfer in the depth direction.

In summary, comparing the temperature fields and melt pool structural dimensions of the four heat sources, both the Gaussian surface heat source and the Gaussian volumetric heat source exhibit certain limitations. The combined heat source demonstrates the strongest overall capability, making it suitable for numerical simulations in scenarios with high requirements for forming quality, ensuring high fidelity in the simulations.

### 4.3. Dynamic Heat Source

Among the four heat source models mentioned above, the combined heat source model provides higher simulation accuracy. However, the SLM forming process involves multiple interdisciplinary fields and various modes of heat transfer, as well as the complex metallurgical process of metal powder undergoing phase transformation in an extremely short time. On one hand, the thermal behavior of the SLM process is extremely complex. In solid materials, laser energy is primarily concentrated on the surface of the material and has low optical penetration, meaning that most of the energy cannot penetrate deeply into the material. In a powder bed, however, the laser does not simply act on the surface of the melt pool; after the light penetrates the powder bed, energy is reflected, absorbed, and re-scattered among the powder particles, resulting in an increased penetration depth of the laser. On the other hand, once the melt pool is formed, the movement of the laser heat source also affects the dynamic morphology of the melt pool and the phase state of the material. During the movement of the laser beam, the front and rear ends may come into contact with the metal material in powder, molten, or solid states [40]. When processing multi-pass, multi-layer formed parts, during the laser scanning of the first melt track, the material in front of the laser beam center consists of porous powder that allows heat flow to penetrate, exhibiting a powdery state. The material at the laser beam center may be in a molten state, while the scanned area and the material behind the laser beam center become dense solid. At this point, heat flow penetration is difficult, and the heat only affects the surface. When scanning the second track, the material the laser contacts may be a mixture of different phases—molten, powdery, and solid states. Additionally, the distribution of these phases is irregular and closely related to factors such as the light source radius, material energy absorption rate, and scanning path.

**Figure 10:**
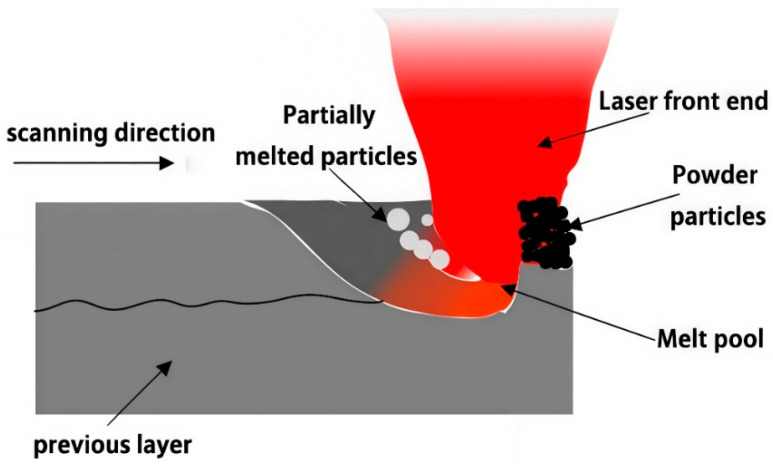
Schematic of laser processing of powder.

Based on the high fidelity of combined heat sources for SLM simulation, there is a significant difference in the thermal flux behavior of the laser heat source at the front and rear ends during the SLM forming process. The traditional method of superimposing combined heat sources is not very accurate, as it simply overlays surface and volumetric heat sources without considering the phase changes occurring during the laser scanning process. Therefore, a new dynamic heat source is designed to clearly simulate the different ways laser energy acts on the front and rear parts of the laser beam. Figure 10 illustrates the laser interaction with the powder during processing. It can be seen that the front scanning area of the heat source consists of porous powder, where there are large gaps between metal powder particles. The laser energy can penetrate through the powder layer and radiate onto the substrate, causing the powder material to melt completely and instantaneously. Thus, the thermal flux distribution in the depth direction of the heat source needs to be emphasized. The front half of the heat source can be modeled using a Gaussian volumetric heat source distribution. In contrast, the material behind the center of the laser beam has transformed into dense solid with only a small amount of partially melted powder particles remaining. A higher temperature laser is required to achieve more thorough melting of the material to improve the quality of the processed part. The heat source energy is applied to the surface of this region in the form of heat flux density. Therefore, a Gaussian surface heat source distribution can be used to approximate the thermal behavior at the rear end of the heat source.

At this point, the energy distribution type of the heat source model can be divided into two parts: the front end and the rear end, which can be described as follows:

(17) $$\left\{\begin{array}{l} q_{f}(x,y,z)=\frac{3AP}{\pi r_{H}^{2}h}\exp(-\frac{3(x^{2}+y^{2}+z^{2})}{r_{H}^{2}}),x\geq 0\\ q_{b}(x,y)=\frac{3AP}{\pi r_{H}^{2}}\exp(-\frac{3(x^{2}+y^{2})}{r_{H}^{2}}),x<0 \end{array}\right.$$

In the formula: $q_{f}(x,y,z)$ represents the heat flux density distribution of the volumetric heat source in the local relative coordinate system $\left(x,y,z\right)$, $q_{b}(x,y)$ represents the heat flux density of the surface distributed heat source at the rear end, *A* indicates the absorption coefficient of the powder material for laser energy, *P* represents the output power of the laser, $r_{H}$ indicates the effective radius of the laser heat source, and h represents the penetration depth of the volumetric distributed heat source.

**Figure 11:**
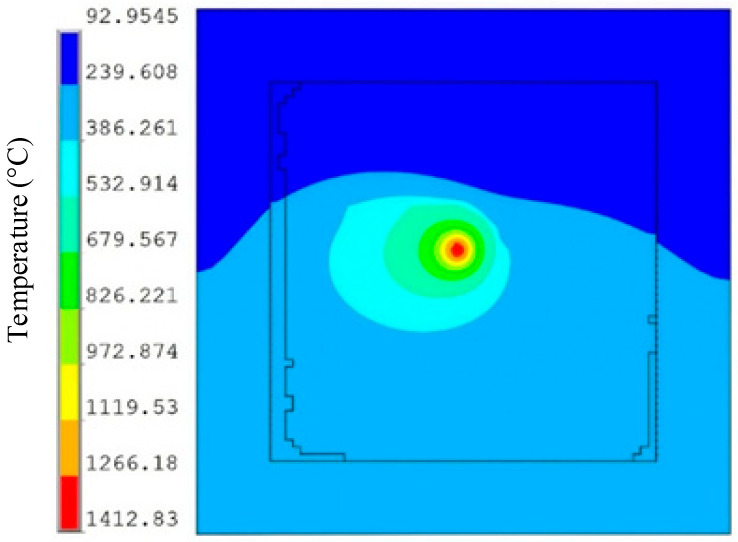
The temperature field cloud map under the dynamic heat source model.

To verify the advantages and disadvantages of the designed dynamic heat source compared to traditional heat sources, all simulation settings are the same as those of the aforementioned heat source models, in order to validate the temperature field characteristics and molten pool structure dimensions under the dynamic heat source model. As shown in Figure 11, the temperature field cloud map at the moment when the laser scans to the midpoint of the fifth melt track is presented. Comparing it with the temperature cloud maps of the first four existing heat sources in Figure 5, it can be observed that the HAZ of the dynamic heat source model is very similar to that of the combined heat source, but the temperatures at all positions are higher than those under the combined heat source. The heat distribution on the surface of the molten pool is more uniform, which can enhance the stability of the molten pool, reduce the occurrence of localized overheating or overcooling areas, and contribute to the formation of a more uniform grain structure, thereby improving the quality of the AM parts.

**Figure 12:**
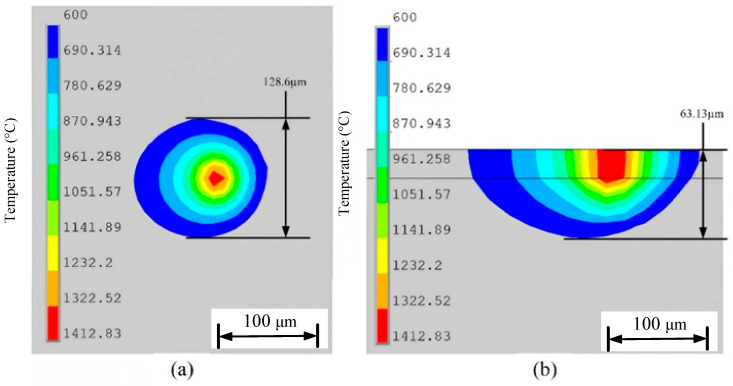
Structure and dimensions of the molten pool under dynamic heat source: (**a**) molten pool width, (**b**) molten pool depth.

Figure 12 shows the molten pool structure and morphology under the dynamic heat source model. In Figure 12a, the surface morphology of the molten pool is displayed, indicating that the molten pool width under this model is 128.6 μm. In comparison, the molten pool width for the combined heat source in Figure 9d is 124.8 μm. The molten pool width under the dynamic heat source is slightly larger, suggesting that the energy distribution pattern of the dynamic heat source allows heat to spread further laterally. Additionally, a more uniform heat input distribution reduces thermal stress caused by temperature gradients during the cooling process. Figure 12b presents the longitudinal cross-section of the molten pool under the dynamic heat source, showing a molten pool depth of 63.13 μm. This represents a significant increase compared to the depths observed in the four heat source models in Figure 9, and is about 17% deeper than the 53.93 μm depth under the combined heat source. The increased depth indicates that more heat penetrates deeper into the material. Unlike the combined heat source, the longitudinal cross-section shows a more pronounced extension of the high-temperature red region at the front end of the heat source, while the red region at the rear end is smaller than that in Figure 9d. This suggests that although the energy distribution pattern at the front and rear ends of the heat source has changed, the effective heat diffusion deeper into the material has not been weakened, demonstrating stronger laser penetration capability.

Excessive pursuit of larger melt pool depth or width dimensions may have negative effects on the simulation. An overly large melt pool size reduces the cooling rate, and the unstable flow of molten metal can cause melt pool spattering, which also impacts the forming quality [41]. Therefore, it is necessary to reasonably control the ratio of melt pool width to depth. A width-to-depth ratio close to two corresponds to a semi-circular melt pool in the conduction–transition regime and is widely regarded as a physically reasonable geometry for stable melting [42]. Table 2 shows the width-to-depth ratios of the melt pools obtained with the four heat-source models. Among them, the dynamic heat source produces a ratio closest to two, indicating a more physically realistic and balanced energy distribution compared with the single heat-source models. This promotes uniform heat transfer within the melt pool and supports stable metallurgical bonding between tracks and layers.

**Figure 13:**
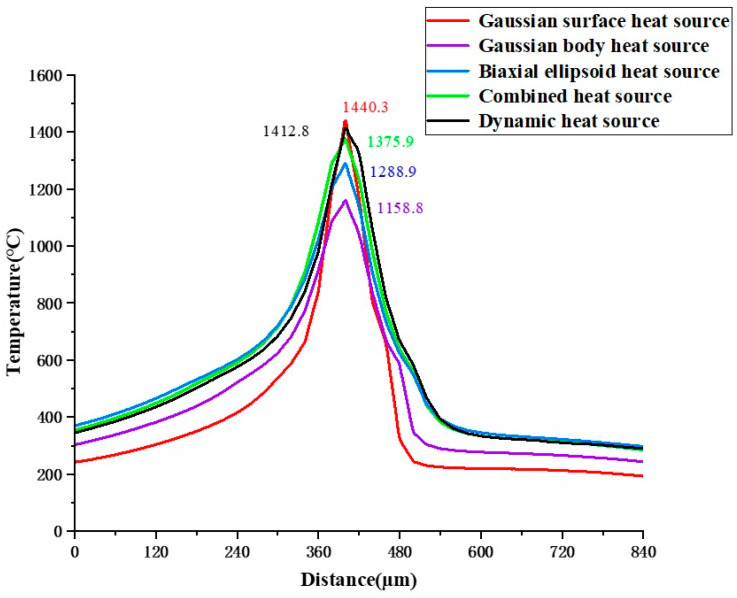
Variation in temperature at node 12920 along the scanning path under different heat source models.

Figure 13 shows the temperature changes at node 12920, located at the midpoint of the fifth melt pool, along the scanning path under different heat source models. This reflects the temperature rise and fall characteristics before and after the heat source moves. From the figure, it can be seen that the Gaussian heat source has the highest peak temperature at this node, reaching 1440.3 °C. The heat input is most concentrated when the heat source is at the center of the path, but the node experiences the fastest heating and cooling rates. The relatively high cooling rate is unfavorable for forming quality. In contrast, the temperature curves for the combined heat source and the dynamic new heat source show smoother temperature rises and falls. The heat source can be maintained for a longer time, which helps reduce thermal stress and minimize the occurrence of stress concentration.

## 5. Experimental Verification

**Figure 14:**
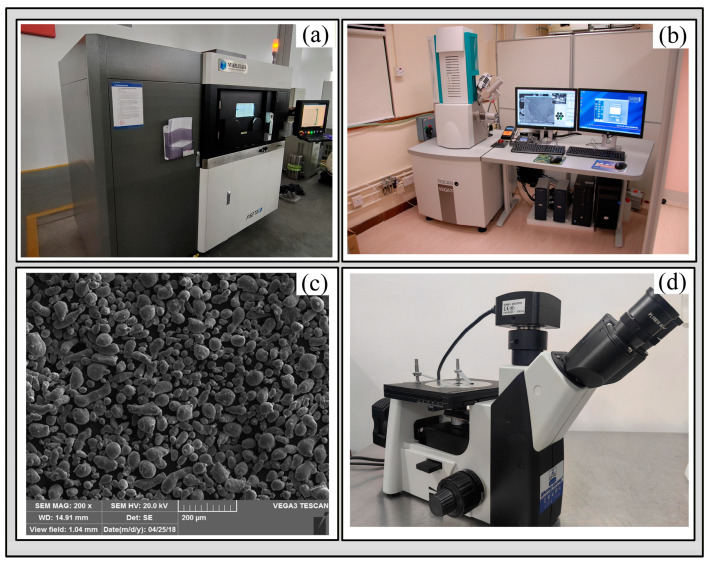
(**a**) The SLM machine (FS273M): (**b**) the Tescan VEGA 3 LMU Scanning Electron Microscope, (**c**) an SEM image of the AlSi_10_Mg powder in use, (**d**) an optical inverted metallurgical microscope (Olympus GX51).

The experiment utilized an FS273M SLM system to fabricate AlSi_10_Mg samples. This equipment is equipped with a fiber laser and a high-precision inert atmosphere control system, as shown in Figure 14a. A substrate preheating device was employed to reduce thermal stress concentration. The original powder was characterized for particle size using a Tescan VEGA 3 LMU scanning electron microscope (SEM), as shown in Figure 14b. The results indicated that the gas-atomized AlSi_10_Mg powder had a particle size distribution ranging from 20 to 50 μm, exhibiting a typical satellite spherical morphology that meets standard requirements, as illustrated in Figure 14c. Surface morphology observation and single-track melt pool width measurements were conducted using an optical inverted metallurgical microscope equipped with a front-facing camera, with a magnification of 100×, as shown in Figure 14d. The process parameter set for the samples included a hatch spacing of 0.12 mm, a scanning speed of 1800 mm/s, and a laser power of 280 W.

**Figure 15:**
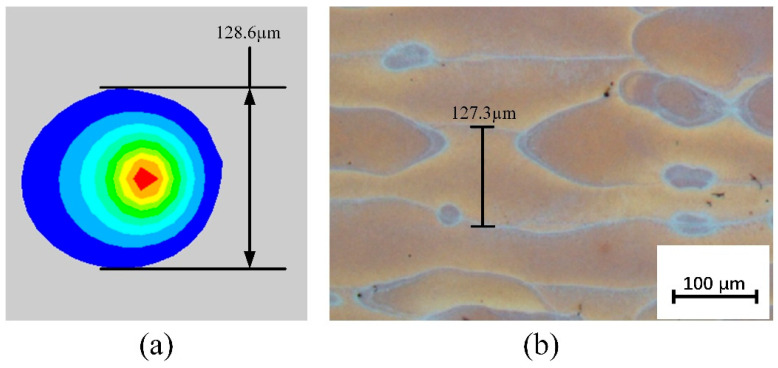
Comparison of melt pool width under dynamic heat sources with experimental results: (**a**) simulation; (**b**) experimental measurement.

**Figure 16:**
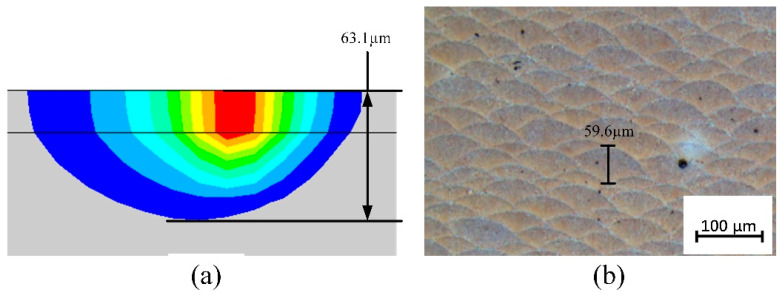
Comparison of melt pool depth under dynamic heat sources with experimental results: (**a**) simulation; (**b**) experimental measurement.

To verify the reliability of the numerical model, Figure 15 shows a comparison between the simulated and experimental results of the melt pool width under a dynamic heat source. Specifically, Figure 15a presents the melt pool surface morphology obtained from numerical simulation, with a measured melt pool width of 128.6 μm; Figure 15b displays the metallographic image of the actual printed sample, from which the melt pool width is measured to be 127.3 μm. The two measurements closely match, with a relative error of only 1.0%, indicating that the simulation model has high accuracy in predicting the lateral dimensions of the melt pool and can effectively reflect the actual melt pool morphology during processing. Figure 16 further compares the melt pool dimensions in the longitudinal cross-section. Figure 16a shows the longitudinal cross-sectional morphology extracted from the simulation, with a depth of 63.1 μm; Figure 16b presents the experimentally measured melt pool depth of 59.6 μm, resulting in a relative error of 5.5%. Although the error in depth is slightly larger than that in width, it remains within an acceptable range, especially considering that melt pool depth is more significantly influenced by factors such as heat accumulation and cooling conditions. These results further validate the overall effectiveness and reliability of the developed model in simulating the melt pool formation process under dynamic heat source conditions.

## 6. Conclusions

This study utilizes the ANSYS APDL simulation module, with AlSi_10_Mg alloy as the processing material, to establish a simulation model for the entire SLM forming process. A dynamic new heat source was designed as the thermal load input model for the SLM simulation. The main conclusions are as follows:

(1) Comparing the temperature cloud map characteristics at the midpoint of the fifth melt track among four heat source models, the volumetric distributed heat source shows a larger HAZ area than the Gaussian surface heat source. However, the peak temperature at the center of the Gaussian surface heat source reaches 1440.34 °C, significantly higher than that of the volumetric distributed heat source. The combined heat source model exhibits an HAZ area larger than that of the Gaussian surface heat source, and the peak temperature at the heat source center is noticeably higher compared to the volumetric distributed heat source model.
(2) By comparing the temperature–time curves at node 12920, it is evident that the double ellipsoid heat source and the combined heat source have stronger thermal accumulation effects. The combined heat source achieves a higher peak temperature of 1375.94 °C. The Gaussian surface heat source results in the shortest molten pool lifetime. Additionally, temperature variations along the scanning path are smoother under the combined heat source model.
(3) Regarding molten pool width, the Gaussian surface heat source yields the largest values, but the combined heat source provides the most uniform energy distribution both laterally and longitudinally. Observing the cross-sectional view along the *Y*-axis at the midpoint of the melt track, the Gaussian surface heat source transfers limited heat in the depth direction. The double ellipsoid heat source has less penetration capability in the powder layer depth compared to the Gaussian volumetric heat source. Under the combined heat source, heat spreads strongly in both lateral and longitudinal directions.
(4) A new dynamic heat source was designed, under which the heat distribution on the melt pool surface is more uniform. Its melt width is 128.6 μm, the largest value compared to the other four heat sources; the melt depth is 63.13 μm, which is about 17% greater than the melt pool depth of the combined heat source model. A physically reasonable melt pool geometry is obtained when the width-to-depth ratio is close to two, and under the proposed dynamic heat source this ratio is 2.03.
(5) Comparison between the simulated melt pool dimensions obtained with the dynamic heat source and the experimentally printed parts shows errors of only 1.0% in melt width and 5.5% in melt depth, demonstrating strong predictive accuracy. These results indicate that the proposed model has high potential for industrial applications, such as process parameter optimization, scan strategy design, and prediction of residual stress and distortion in SLM manufacturing of AlSi_10_Mg components.

Although several necessary physical simplifications are adopted in the present model, such as neglecting explicit melt pool flow effects and treating the powder bed as a continuous medium, these assumptions have been widely demonstrated to be effective and reliable for part-scale thermo-mechanical analysis. Future work will focus on extending the proposed dynamic heat source to multi-layer and multi-track simulations, incorporating temperature-dependent absorptivity, and developing thermo–mechanical–microstructural coupled models. In addition, systematic validation will be conducted for a broader range of materials, including aluminum alloys, titanium alloys, and nickel-based superalloys, as well as more complex scanning strategies, to further promote the application of the proposed model in industrial-scale process optimization and digital manufacturing platforms.

## Figures and Tables

**Table 1:** Numerical solver and time-stepping settings for SLM thermal simulation.

Category	Setting	Value
Analysis type	Transient thermal	ANTYPE = 4
Transient option	Full transient	TRNOPT = FULL
Nonlinear solver	Newton–Raphson	NROPT = FULL
Stabilization	Line search	LNSRCH = ON
Thermal transient	Enabled	TIMINT, ON, THERM
Time integration	First-order	TINTP = 1
Laser step size (PD)	In-plane mesh size	0.02 mm
Scanning speed	*v*	1800 mm/s
Exposure time per step	$T_{1}=PD/v$	$1.11\;\times \;10^{-5}$ s
Sub-steps per laser step	–	2
Time increment	$\Delta t={\;T}_{1}/2$	5.56 μs
Automatic time stepping	Enabled	AUTOTS = 1
Max iterations per sub-step	–	NEQIT = 200
Convergence stop	Enabled	NCNV
Cooling stage sub-steps	–	NSUBST = 20
Initial powder temperature	–	25 °C
Initial substrate temperature	–	60 °C
Conv	–	100 W/m^3^

**Table 2:** Width-to-depth ratios of molten pools under different heat source models.

Type of Heat Source	Melt Pool Width (μm)	Melt Pool Depth (μm)	Width-to-Depth Ratio
Gaussian surface heat source	126.8	29.41	4.31
Rotating Gaussian body heat source	118.6	38.02	3.12
Biaxial ellipsoid heat source	115.2	42.32	2.72
Combined heat source	124.8	53.93	2.31
Dynamic heat source	128.6	63.13	2.03

## Data Availability

The original contributions presented in this study are included in the article/Supplementary Materials. Further inquiries may be directed to the corresponding authors.

## Supplementary Materials

The Combined Heat Source Related Code can be downloaded at https://www.mdpi.com/article/10.3390/ma19030480/s1.
