# Supplementary material for: A Comparative Study and Introduction of a New Heat Source Model for the Macro-Scale Numerical Simulation of Selective Laser Melting Technology
Source: Materials (Basel). 2026 Jan 25;19(3):480. doi: 10.3390/ma19030480 (PMC12897802; doi:10.3390/ma19030480)
Supplement: Supplementary file 1 [file materials-19-00480-s001.zip › materials-4080268-supplementary.pdf]

Attached file

Pseudocode APDL

```
/REPLOT

/UNITS,SI
/FILNAME,5HEAT,1
/TITLE,FEM FOR SLM Laser

/NERR,0
KEYW,PR_SGVOF,1
/uis,msgpop,3
/PREP7
TOFFST,273
*SET,BEDHIGH,3*30E-6
*SET,LENGTH,1040E-6
*SET,WIDE,1040E-6
*SET,THICKNESS,-200E-6
*SET,BASEHIGH,-200E-6
*SET,LSIZE,20E-6
*SET,LSIZE2,10E-6
Ni=0.07
Yi=0.1
D=40e-6
ah=D/2
chf=2*ah
chb=4*ah
bh=50e-6
rf=2*chf/(chf+chb)
rb=2*chb/(chf+chb)
fyl=chf/ah
byl=chb/ah
fbzw=bh/ah
*SET,R,33E-6
*SET,P,340
*SET,H,30E-6
*SET,A,0.11
*SET,V,1.2
*SET,PI,3.14
*SET,PD,40E-6
*SET,T1,PD/V
*SET,T2,20E-6
*SET,TT,T1
*SET,S,120E-6
*SET,NUMCONV,100


/SOLU
```

```
ANTYPE,4
TRNOPT,FULL
NROPT,FULL,,ON
Timint,on,therm
TINTP,,,,1
LNSRCH,ON
TSRES,ERASE
AUTOTS,1
NEQIT,200
KBC,1
NCNV,2,10E10
ALLSEL,ALL
LOCAL,11,0,DISX,DISY,DISZ, , , ,,,
NSEL,S,LOC,X,0,10*R
NSEL,R,LOC,Y,-10*R,10*R
NSEL,R,LOC,Z,0,30E-6
CM,FRONT,NODE

LOCAL,13,0,DISX,DISY,DISZ,,,,,,
*DEL,TALBENAME
*DEL,TALBEMTID
*DEL,TABLE_ROW1
*DEL,TABLE_ROW2
*DEL,TABLE_ROW3
*DEL,TABLE_ROW4
*DEL,TABLECSYS
*DEL,TEMPF

*SET,TABLENAME,'TFRONT'
*DIM,TABLE_ROW1,,1
*DIM,TABLE_ROW2,,1
*DIM,TABLE_ROW3,,1
*DIM,TABLE_ROW4,,1
*SET,TABLE_ROW1(1),A
*SET,TABLE_ROW2(1),P
*SET,TABLE_ROW3(1),H
*SET,TABLE_ROW4(1),R
*SET,TABLECSYS,13

*DIM,%TABLENAME%,TABLE,6,26,1,,,,%TABLECSYS%

! Begin of equation: 2*A*P/(3.14*H*R^2)*exp(-2({X}^2+{Y}^2)/R^2)
*SET,%TABLENAME%(0,0,1),0.0, -999
*SET,%TABLENAME%(2,0,1),0.0
*SET,%TABLENAME%(3,0,1),%TABLE_ROW1(1)%
*SET,%TABLENAME%(4,0,1),%TABLE_ROW2(1)%
*SET,%TABLENAME%(5,0,1),%TABLE_ROW3(1)%
*SET,%TABLENAME%(6,0,1),%TABLE_ROW4(1)%
*SET,%TABLENAME%(0,1,1), 1.0, -1, 0, 2, 0, 0, 17
*SET,%TABLENAME%(0,2,1), 0.0, -2, 0, 1, -1, 3, 17
*SET,%TABLENAME%(0,3,1),   0, -1, 0, 1, -2, 3, 18
```

```
*SET,%TABLENAME%(0,4,1), 0.0, -2, 0, 3.14, 0, 0, 19
*SET,%TABLENAME%(0,5,1), 0.0, -3, 0, 1, -2, 3, 19
*SET,%TABLENAME%(0,6,1), 0.0, -2, 0, 2, 0, 0, 20
*SET,%TABLENAME%(0,7,1), 0.0, -4, 0, 1, 20, 17, -2
*SET,%TABLENAME%(0,8,1),  0.0, -2, 0, 1, -3, 3, -4
*SET,%TABLENAME%(0,9,1), 0.0, -3, 0, 1, -1, 4, -2
*SET,%TABLENAME%(0,10,1), 0.0, -1, 0, 0, 0, 0, 0
*SET,%TABLENAME%(0,11,1), 0.0, -2, 0, 1, 0, 0, -1
*SET,%TABLENAME%(0,12,1), 0.0, -4, 0, 1, -1, 2, -2
*SET,%TABLENAME%(0,13,1), 0.0, -1, 0, 2, 0, 0, 2
*SET,%TABLENAME%(0,14,1), 0.0, -2, 0, 1, 2, 17, -1
*SET,%TABLENAME%(0,15,1), 0.0, -1, 0, 2, 0, 0, 3
*SET,%TABLENAME%(0,16,1), 0.0, -5, 0, 1, 3, 17, -1
*SET,%TABLENAME%(0,17,1), 0.0, -1, 0, 1, -2, 1, -5
*SET,%TABLENAME%(0,18,1), 0.0, -2, 0, 2, 0, 0, -1
*SET,%TABLENAME%(0,19,1), 0.0, -5, 0, 1, -2, 3, -1
*SET,%TABLENAME%(0,20,1),0.0, -1, 0, 1, -4, 3, -5
*SET,%TABLENAME%(0,21,1), 0.0, -2, 0, 2, 0, 0, 20
*SET,%TABLENAME%(0,22,1), 0.0, -4, 0, 1, 20, 17, -2
*SET,%TABLENAME%(0,23,1), 0.0, -2, 0, 1, -1, 4, -4
*SET,%TABLENAME%(0,24,1), 0.0, -1, 7, 1, -2, 0, 0
*SET,%TABLENAME%(0,25,1), 0.0, -2, 0, 1, -3, 3, -1
*SET,%TABLENAME%(0,26,1),  0.0, 99, 0, 1, -2, 0, 0
! End of equation: 2*A*P/(3.14*H*R^2)*exp(-2({X}^2+{Y}^2)/R^2)
!-->
```
